# 4-Acetyl­pyridinium iodide

**DOI:** 10.1107/S1600536810021860

**Published:** 2010-06-16

**Authors:** Jie Xu, Xue-qun Fu

**Affiliations:** a Ordered Matter Science Research Center, Southeast University, Nanjing 210096, People’s Republic of China

## Abstract

In the title compound, C_7_H_8_NO^+^·I^−^, N—H⋯I hydrogen bonding and π–π stacking inter­actions [centroid–centroid distance = 5.578 (4) Å] stabilize the structure.

## Related literature

For background to phase transition materials, see: Li *et al.* (2008 ▶); Zhang *et al.* (2009 ▶). For 4-acetyl­pyridine as a ligand in coordination compounds, see: Steffen & Palenik (1977 ▶); Pang *et al.* (1994 ▶). For other structures involving 4-acetyl­pyridine, see: Fu (2009*a* ▶,*b* ▶); Majerz *et al.* (1991 ▶).
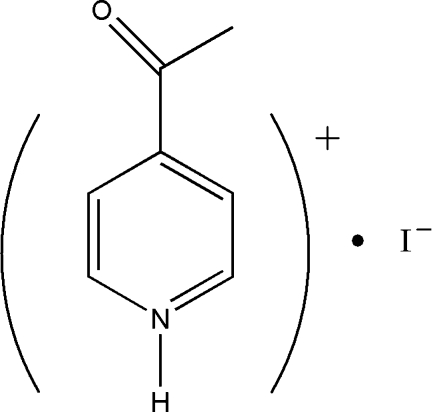


## Experimental

### 

#### Crystal data

C_7_H_8_NO^+^·I^−^
*M* *_r_* = 249.04
Monoclinic, 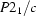

*a* = 8.5144 (17) Å
*b* = 5.0926 (10) Å
*c* = 21.714 (6) Å
β = 111.37 (3)°
*V* = 876.8 (3) Å^3^
*Z* = 4
Mo *K*α radiation
μ = 3.59 mm^−1^
*T* = 298 K
0.40 × 0.30 × 0.20 mm

#### Data collection

Rigaku SCXmini diffractometer
Absorption correction: multi-scan (*CrystalClear*; Rigaku, 2005 ▶) *T*_min_ = 0.286, *T*_max_ = 0.488
8420 measured reflections
2006 independent reflections
1805 reflections with *I* > 2σ(*I*)
*R*_int_ = 0.039

#### Refinement

*R*[*F* ^2^ > 2σ(*F* ^2^)] = 0.040
*wR*(*F* ^2^) = 0.124
*S* = 0.90
2006 reflections
91 parameters
H-atom parameters constrained
Δρ_max_ = 0.70 e Å^−3^
Δρ_min_ = −0.62 e Å^−3^

### 

Data collection: *CrystalClear* (Rigaku, 2005 ▶); cell refinement: *CrystalClear*; data reduction: *CrystalClear*; program(s) used to solve structure: *SHELXS97* (Sheldrick, 2008 ▶); program(s) used to refine structure: *SHELXL97* (Sheldrick, 2008 ▶); molecular graphics: *SHELXTL* (Sheldrick, 2008 ▶); software used to prepare material for publication: *PRPKAPPA* (Ferguson, 1999 ▶).

## Supplementary Material

Crystal structure: contains datablocks I, global. DOI: 10.1107/S1600536810021860/jh2163sup1.cif

Structure factors: contains datablocks I. DOI: 10.1107/S1600536810021860/jh2163Isup2.hkl

Additional supplementary materials:  crystallographic information; 3D view; checkCIF report

## Figures and Tables

**Table 1:** Hydrogen-bond geometry (Å, °)

*D*—H⋯*A*	*D*—H	H⋯*A*	*D*⋯*A*	*D*—H⋯*A*
N1—H1*A*⋯I1^i^	0.86	2.67	3.456 (6)	153

Symmetry code: (i) 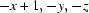.

**Fig. 1.**
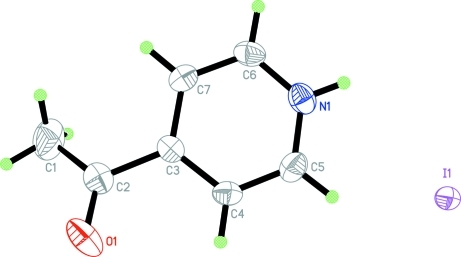
The molecular structure of the title compound, with the atomic numbering scheme. Displacement ellipsoids are drawn at the 30% probability level, and all H atoms have been omitted for clarity.

**Fig. 2.**
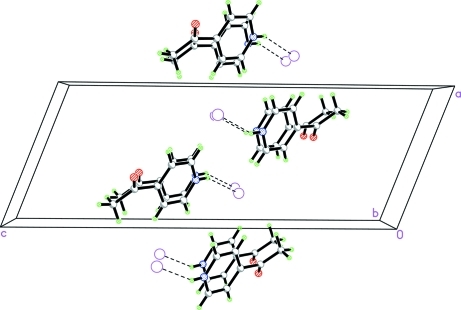
A view of the packing of the title compound, stacking along the b axis. Dashed lines indicate hydrogen bonds.

## supplementary crystallographic information

### Comment

As a continuation of our study of phase transition materials, including organic ligands (Li *et al.*, 2008), metal-organic coordination compounds (Zhang *et al.*, 2009), organic-inorganic hybrids, we studied the dielectric properties of the title compound, unfortunately, there was no distinct anomaly observed from 93 K to 350 K, (subliming above 388 K), suggesting that this compound should be not a real ferroelectrics or there may be no distinct phase transition occurred within the measured temperature range.In this article, the crystal structure of the title compound has been presented.

4-Acetylpyridine may be used as a ligand in coordination compounds *e.g.* with Zn (Steffen & Palenik, 1977) or Ni (Pang *et al.*, 1994). The crystal structures of 4-acetylpyridine together with pentachlorophenol (Majerz *et al.* 1991) andinorganic acids are also known *e.g.* with sulfuric acid (Fu, 2009b) and perchloric acid (Fu, 2009a).

The asymmetric unit of the title compound is built up from an protonated 4-acetylpyridinium cation wherein the acetyl group deviates 28.0 (5)°from the plane formed by the non-hydrogen atoms of the pyridine ring and a I^-^ anion (Fig. 1). The C1—C2—O1 bond angle and O1—C2—C3—C4 torsion angle are 122.6 (8)ånd 27.8 (9)°, respectively. N—H···I hydrogen bonding (N···I distance 3.456 (6) Å) and π-π stacking interaction with the adjacent interplanar spacing of 5.578 (4)Å make great contribution to the stability of the crystal structure.

### Experimental

1.19 g(10 mmol) 4-acetylpyridine was firstly dissolved in 50 ml e thanol, to which hydroiodic acid aqueous solution(40%, *w*/*w*) was then added until the solution became acidic under stirring.Single crystals of (I) were prepared by slow evaporation at room temperature of the acidic solution after 3 days.

### Refinement

Positional parameters of all the H atoms were calculated geometrically and were allowed to ride on the C and N atoms to which they are bonded, with *U*_iso_(H) = 1.2*U*_eq_(C),*U*_iso_(H) = 1.5*U*_eq_(C) for methyl group and *U*_iso_(H) = 1.2*U*_eq_(N).

### Crystal data

**Table:**

C_7_H_8_NO^+^·I^−^	*F*(000) = 472
*M**_r_* = 249.04	*D*_x_ = 1.887 Mg m^−^^3^
Monoclinic, *P*2_1_/*c*	Mo *K*α radiation, λ = 0.71073 Å
Hall symbol: -P 2ybc	Cell parameters from 4122 reflections
*a* = 8.5144 (17) Å	θ = 3.0–27.6°
*b* = 5.0926 (10) Å	µ = 3.59 mm^−^^1^
*c* = 21.714 (6) Å	*T* = 298 K
β = 111.37 (3)°	Prism, colourless
*V* = 876.8 (3) Å^3^	0.40 × 0.30 × 0.20 mm
*Z* = 4	

### Data collection

**Table:**

Rigaku SCXmini diffractometer	2006 independent reflections
Radiation source: fine-focus sealed tube	1805 reflections with *I* > 2σ(*I*)
graphite	*R*_int_ = 0.039
Detector resolution: 13.6612 pixels mm^-1^	θ_max_ = 27.5°, θ_min_ = 3.8°
ω scans	*h* = −11→11
Absorption correction: multi-scan (*CrystalClear*; Rigaku, 2005)	*k* = −6→6
*T*_min_ = 0.286, *T*_max_ = 0.488	*l* = −28→27
8420 measured reflections	

### Refinement

**Table:**

Refinement on *F*^2^	Primary atom site location: structure-invariant direct methods
Least-squares matrix: full	Secondary atom site location: difference Fourier map
*R*[*F*^2^ > 2σ(*F*^2^)] = 0.040	Hydrogen site location: inferred from neighbouring sites
*wR*(*F*^2^) = 0.124	H-atom parameters constrained
*S* = 0.90	*w* = 1/[σ^2^(*F*_o_^2^) + (0.0645*P*)^2^ + 6.0704*P*] where *P* = (*F*_o_^2^ + 2*F*_c_^2^)/3
2006 reflections	(Δ/σ)_max_ < 0.001
91 parameters	Δρ_max_ = 0.70 e Å^−^^3^
0 restraints	Δρ_min_ = −0.62 e Å^−^^3^

### Special details

**Table:**

Geometry. All e.s.d.'s (except the e.s.d. in the dihedral angle between two l.s. planes) are estimated using the full covariance matrix. The cell e.s.d.'s are taken into account individually in the estimation of e.s.d.'s in distances, angles and torsion angles; correlations between e.s.d.'s in cell parameters are only used when they are defined by crystal symmetry. An approximate (isotropic) treatment of cell e.s.d.'s is used for estimating e.s.d.'s involving l.s. planes.
Refinement. Refinement of *F*^2^ against ALL reflections. The weighted *R*-factor *wR* and goodness of fit *S* are based on *F*^2^, conventional *R*-factors *R* are based on *F*, with *F* set to zero for negative *F*^2^. The threshold expression of *F*^2^ > σ(*F*^2^) is used only for calculating *R*-factors(gt) *etc*. and is not relevant to the choice of reflections for refinement. *R*-factors based on *F*^2^ are statistically about twice as large as those based on *F*, and *R*- factors based on ALL data will be even larger.

### Fractional atomic coordinates and isotropic or equivalent isotropic displacement parameters (Å^2^)

**Table:**

	*x*	*y*	*z*	*U*_iso_*/*U*_eq_	
I1	0.78163 (5)	0.05660 (9)	0.07248 (2)	0.05081 (18)	
O1	0.3686 (9)	1.1056 (11)	0.2117 (3)	0.0769 (18)	
N1	0.3294 (8)	0.3813 (11)	0.0580 (3)	0.0531 (13)	
H1A	0.3405	0.2717	0.0297	0.064*	
C2	0.2855 (10)	0.9025 (15)	0.2014 (3)	0.0546 (16)	
C7	0.1674 (9)	0.5509 (14)	0.1144 (3)	0.0512 (15)	
H7A	0.0675	0.5531	0.1226	0.061*	
C5	0.4569 (9)	0.5415 (15)	0.0887 (4)	0.0552 (16)	
H5A	0.5549	0.5357	0.0792	0.066*	
C6	0.1850 (9)	0.3817 (13)	0.0688 (4)	0.0528 (16)	
H6A	0.0978	0.2686	0.0455	0.063*	
C3	0.2973 (8)	0.7194 (12)	0.1488 (3)	0.0412 (12)	
C4	0.4428 (8)	0.7145 (13)	0.1342 (3)	0.0478 (14)	
H4A	0.5307	0.8293	0.1556	0.057*	
C1	0.1781 (13)	0.832 (3)	0.2385 (4)	0.093 (3)	
H1B	0.1832	0.9683	0.2697	0.140*	
H1C	0.2171	0.6700	0.2617	0.140*	
H1D	0.0638	0.8111	0.2085	0.140*	

### Atomic displacement parameters (Å^2^)

**Table:**

	*U*^11^	*U*^22^	*U*^33^	*U*^12^	*U*^13^	*U*^23^
I1	0.0462 (3)	0.0495 (3)	0.0573 (3)	−0.00662 (19)	0.01939 (19)	−0.00989 (18)
O1	0.122 (5)	0.044 (3)	0.059 (3)	−0.005 (3)	0.026 (3)	−0.008 (2)
N1	0.071 (4)	0.041 (3)	0.049 (3)	0.000 (3)	0.024 (3)	−0.001 (2)
C2	0.062 (4)	0.054 (4)	0.042 (3)	0.007 (3)	0.012 (3)	0.005 (3)
C7	0.044 (3)	0.055 (4)	0.056 (4)	−0.002 (3)	0.020 (3)	0.004 (3)
C5	0.054 (4)	0.057 (4)	0.063 (4)	0.001 (3)	0.031 (3)	0.005 (3)
C6	0.052 (4)	0.039 (3)	0.061 (4)	−0.008 (3)	0.013 (3)	−0.002 (3)
C3	0.049 (3)	0.036 (3)	0.035 (3)	0.003 (2)	0.010 (2)	0.006 (2)
C4	0.047 (3)	0.045 (3)	0.049 (3)	−0.010 (3)	0.014 (3)	0.000 (3)
C1	0.094 (7)	0.140 (10)	0.057 (5)	0.014 (7)	0.040 (5)	−0.003 (6)

### Geometric parameters (Å, °)

**Table:**

O1—C2	1.227 (9)	C5—C4	1.362 (10)
N1—C5	1.327 (9)	C5—H5A	0.9300
N1—C6	1.332 (10)	C6—H6A	0.9300
N1—H1A	0.8600	C3—C4	1.385 (9)
C2—C1	1.468 (11)	C4—H4A	0.9300
C2—C3	1.506 (9)	C1—H1B	0.9600
C7—C6	1.363 (10)	C1—H1C	0.9600
C7—C3	1.383 (9)	C1—H1D	0.9600
C7—H7A	0.9300		
			
C5—N1—C6	123.3 (6)	C7—C6—H6A	120.6
C5—N1—H1A	118.3	C7—C3—C4	118.1 (6)
C6—N1—H1A	118.3	C7—C3—C2	122.1 (6)
O1—C2—C1	122.6 (8)	C4—C3—C2	119.8 (6)
O1—C2—C3	117.9 (7)	C5—C4—C3	120.0 (6)
C1—C2—C3	119.5 (8)	C5—C4—H4A	120.0
C6—C7—C3	120.4 (6)	C3—C4—H4A	120.0
C6—C7—H7A	119.8	C2—C1—H1B	109.5
C3—C7—H7A	119.8	C2—C1—H1C	109.5
N1—C5—C4	119.3 (6)	H1B—C1—H1C	109.5
N1—C5—H5A	120.3	C2—C1—H1D	109.5
C4—C5—H5A	120.3	H1B—C1—H1D	109.5
N1—C6—C7	118.8 (6)	H1C—C1—H1D	109.5
N1—C6—H6A	120.6		
			
C6—N1—C5—C4	0.8 (11)	C1—C2—C3—C7	27.6 (10)
C5—N1—C6—C7	−1.3 (11)	O1—C2—C3—C4	27.8 (9)
C3—C7—C6—N1	0.2 (10)	C1—C2—C3—C4	−151.5 (7)
C6—C7—C3—C4	1.4 (10)	N1—C5—C4—C3	0.9 (10)
C6—C7—C3—C2	−177.8 (6)	C7—C3—C4—C5	−2.0 (9)
O1—C2—C3—C7	−153.0 (7)	C2—C3—C4—C5	177.2 (6)

### Hydrogen-bond geometry (Å, °)

**Table:**

*D*—H···*A*	*D*—H	H···*A*	*D*···*A*	*D*—H···*A*
N1—H1A···I1^i^	0.86	2.67	3.456 (6)	153

Symmetry codes: (i) −*x*+1, −*y*, −*z*.
